# Mutant p53 Associates with Human Equilibrative Nucleoside 1 Upregulation and Better Response to Adjuvant Gemcitabine in Intrahepatic Cholangiocarcinoma Patients

**DOI:** 10.3390/ijms26115259

**Published:** 2025-05-30

**Authors:** Marzia Deserti, Valeria Relli, Andrea Palloni, Francesco Vasuri, Deborah Malvi, Alessio Degiovanni, Simone Rimedio, Chiara Delbaldo, Chiara Deiana, Giovanni Brandi, Simona Tavolari

**Affiliations:** 1 Department of Medical and Surgical Sciences, University of Bologna, 40126 Bologna, Italy; marzia.deserti2@unibo.it (M.D.); valeria.relli@unibo.it (V.R.); francesco.vasuri2@unibo.it (F.V.); simone.rimedio2@unibo.it (S.R.); chiara.delbaldo2@unibo.it (C.D.); chiarad.deiana2@unibo.it (C.D.); giovanni.brandi@unibo.it (G.B.); 2 Medical Oncology, IRCCS Azienda Ospedaliero-Universitaria di Bologna, 40138 Bologna, Italy; andrea.palloni@aosp.bo.it; 3 Pathology Unit, Santa Maria delle Croci Ravenna Hospital, 48121 Ravenna, Italy; 4 Pathology Unit, IRCCS Azienda Ospedaliero-Universitaria di Bologna, 40138 Bologna, Italy; deborah.malvi@aosp.bo.it (D.M.); alessio.degiovanni@gmail.com (A.D.)

**Keywords:** intrahepatic cholangiocarcinoma, hENT-1, p53 status, adjuvant gemcitabine

## Abstract

The prognostic and predictive role of the human equilibrative nucleoside transporter 1 (hENT-1) has emerged in different cancer types, including intrahepatic cholangiocarcinoma (iCCA), but the mechanisms regulating its expression are poorly understood. Here, we investigated the link between p53 status and hENT-1 regulation in 38 iCCA patients and cell line models; the predictive role of p53 status in response to adjuvant gemcitabine was also investigated. A positive association between mutant p53 cells and hENT-1 expression was observed in iCCA tissue samples; furthermore, patients receiving adjuvant gemcitabine and expressing mutant p53 cells > 4% in tumor tissue had a longer disease-free survival (DFS) than patients expressing mutant p53 cells ≤ 4% (median 18.5 vs. 6 months, *p* = 0.0229). In iCCA cell line models, transient knockdown of mutant p53 resulted in a decrease in hENT-1 mRNA and protein expression; similarly, restoration of wild-type p53 function induced a significant reduction in hENT-1 mRNA and protein expression. Overall, these findings support a role of p53 status in the regulation of hENT-1 expression, suggesting an opposite effect (activating versus repressive) of mutant and wild-type p53 protein. Furthermore, although the present study should be considered as preliminary, our findings suggest a predictive role of p53 status in iCCA patients treated with gemcitabine, thus deserving future investigations in additional cohorts of cancer patients.

## 1. Introduction

Intrahepatic cholangiocarcinoma (iCCA) is a malignancy of the bile ducts within the liver with poor prognosis; currently a limited number of patients (10–20%) are candidates for surgery, the only potentially curative option [1]. For more than a decade, gemcitabine plus cisplatin has represented the standard of care in a first-line setting in patients with advanced or metastatic disease, the majority of diagnosed iCCA cases [2]. Very recently, gemcitabine in combination with cisplatin and the PD-L1 inhibitor Durvalumab has been approved as a new standard of care in this setting [3]. Despite these therapeutic advances, the prognosis of iCCA still remains discouraging and the identification of prognostic and predictive biomarkers able to improve the clinical outcome of these patients therefore represents an intense area of investigation.

In this context, we previously reported the prognostic and predictive role of equilibrative nucleoside transporter 1 (hENT-1) expression/localization in CCA patients [4,5]. hENT-1 is the most abundant membrane nucleoside transporter in human cells and mediates the cellular uptake of purine/pyrimidine nucleosides that serve as nucleotide precursors for nucleic acid biosynthesis and as coenzymes for many metabolic reactions [6]. Furthermore, this transporter is known to regulate the cellular uptake of some nucleoside-analogue drugs commonly employed in clinical practice for cancer treatment, including gemcitabine [6]. Despite these central biological roles, the molecular mechanisms regulating hENT-1 expression in cancer patients have been less investigated.

Mutations of the TP53 gene are the most frequent genomic alterations detected in human cancers and have been implicated also in iCCA carcinogenesis, occurring with a frequency from 18% to 43% of cases [7]. Most TP53 alterations are missense mutations occurring within the DNA binding domain, with the R175, G245, R248, R249, R273, and R282 sites representing the most frequent mutational hotspots [8]. Typically, encoded mutant p53 proteins lose canonical wild-type p53 functions and gain oncogenic properties that promote cancer development and progression [9]. Recently, the regulation of genes involved in nucleotide metabolism has been reported as a novel molecular mechanism contributing to the oncogenic properties of mutant p53 [10].

In light of the central role of hENT-1 in regulating the cellular uptake of nucleosides and nucleoside-analogue drugs, in the present study we investigated the possible link between p53 status and hENT-1 regulation in iCCA patients and cell line models; the predictive role of p53 status in the response to adjuvant gemcitabine of iCCA patients was also investigated.

## 2. Results

### 2.1. Mutant p53 Associates with High hENT-1 Expression and Membrane Localization in iCCA Tissue Samples

We firstly evaluated the association between mutant p53 and hENT-1 protein expression in the tumor tissue from 38 iCCA patients; their main clinical–pathological variables are summarized in Table 1. The cohort consisted of 18 men and 20 women, with a median age at surgery of 61 years; none of them received radiotherapy or chemotherapy before surgery. Macroscopically, all iCCAs were of the mass-forming type; tumor grade was 1 in 9 (23.7%) cases, 2 in 8 (21%) cases, 3 in 13 (34.2%) cases and 4 in 3 (7.9) cases.

**Figure 1:**
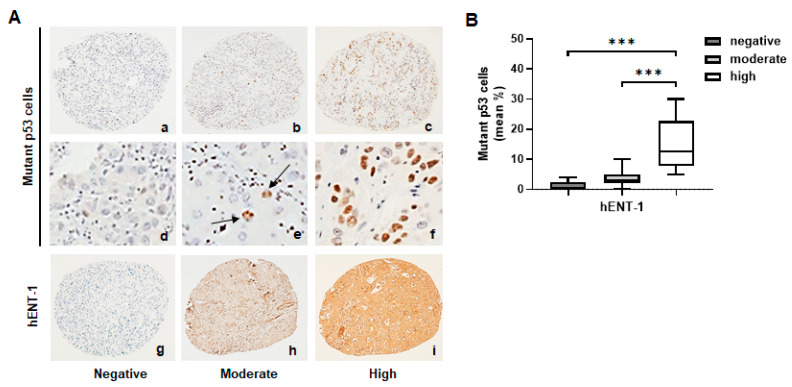
(**A**) Representative IHC images of mutant p53 cells (a–f) and negative, moderate, and high hENT-1 expression (g–i) in iCCA tissue samples. Black arrows show mutant p53 positive cells. Panels (a–c,g–i): magnification 20×; (d–f): magnification 40×. (**B**) Box plot analysis of mutant p53 cells in iCCA samples according to negative, moderate, and high hENT-1 expression in tumor tissue. *** *p* < 0.0001.

**Figure 2:**
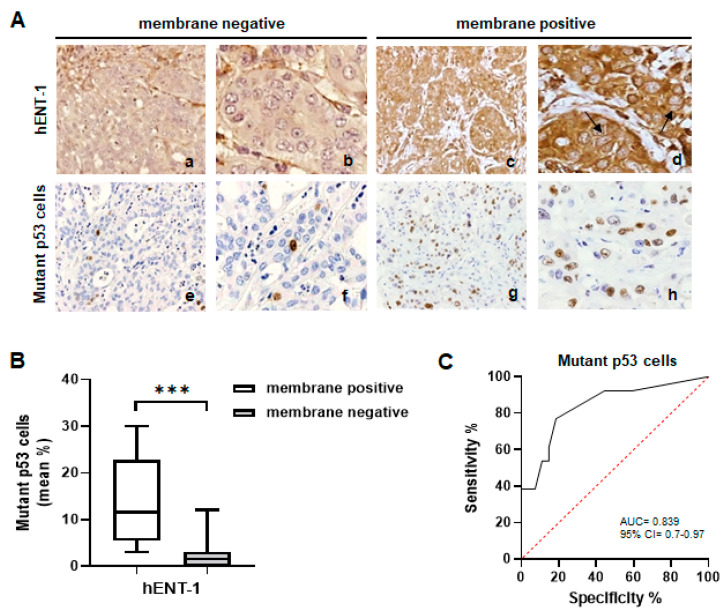
(**A**) Representative IHC images showing the correlation between membrane negative and membrane positive hENT-1 expression (a–d) and mutant p53 cells (e–h) in iCCA tissue samples. Black arrows show membrane hENT-1 positivity in cancer cells (a,e,c,g): magnification 20×; (b,f,d,h): magnification 40×. (**B**) Box plot analysis of mutant p53 cells in iCCA samples according to hENT-1 membrane positive and membrane negative expression in tumor tissue. *** *p* < 0.0001. (**C**) ROC curve showing the 4% cut-off value of mutant p53 cells able to predict hENT-1 membrane positivity with 77% sensitivity and 81% specificity (AUC 0.839).

At IHC analysis, mutant p53 cells were absent in 11 (29%) of cases, ≤10% in 17 (44.7%) cases, and >10% in the remaining 10 (26.3%) cases (Figure 1A(a–f)). Stratifying patients according to hENT-1 expression, immunoreactivity was absent in 14 (37%) cases, moderate in 12 (31.5%), and high in 12 (31.5%) cases (Figure 1A(g–i)). Among the whole cohort of patients, a positive association between hENT-1 expression and mutant p53 cells was observed. The mean percentage of mutant p53 cells was 1.7% in patients negative for hENT-1 expression, 3.4% in patients with moderate hENT-1 expression, and 13.1% in patients with high hENT-1 expression (Figure 1B). Although the mean percentage of mutant p53 cells did not significantly differ between hENT-1 negative and moderate patients’ subgroups, the difference became significant when these subgroups were compared to the subgroup of high hENT-1 patients (hENT-1 negative vs. hENT-1 high, *p* < 0.0001; hENT-1 moderate vs. hENT-1 high, *p* < 0.0001). Further IHC analysis among the 24 patients positive for hENT-1 expression showed a concomitant membrane/cytoplasmic staining in 13 (54.2%) cases, while 11 (45.8%) cases showed only a cytoplasmic positivity (Figure 2A(a–d)). Notably, a higher mean percentage of mutant p53 cells was detected in membrane hENT-1 positive patients compared to membrane hENT-1 negative (11% vs. 3%, *p* < 0.0001) (Figure 2A(e–h),B). ROC curve indicated that a 4% cut-off value of mutant p53 cells was able to predict hENT-1 membrane positivity with 77% sensitivity and 81% specificity (AUC 0.839, Figure 2C).

**Figure 3:**
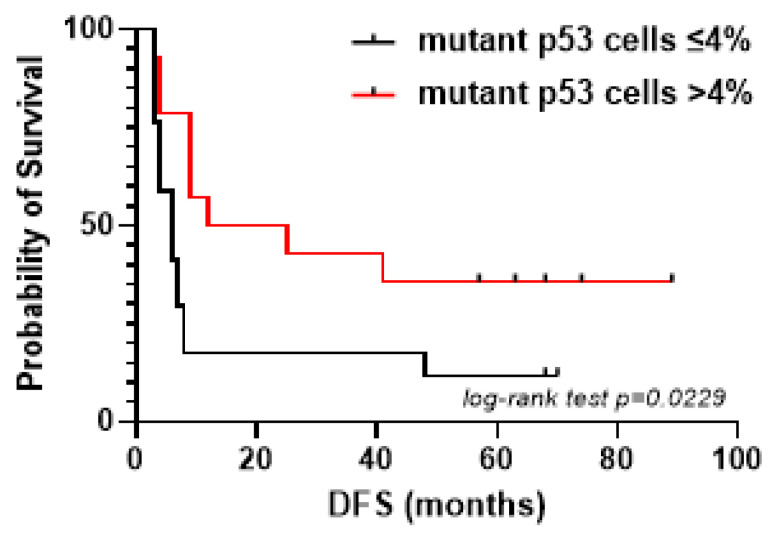
DFS curves in iCCA patients receiving adjuvant gemcitabine stratified according to mutant p53 cells ≤ 4% and >4% in tumor tissue (*p* = 0.0229).

In detail, as reported in Table 2, a mean percentage of mutant p53 cells > 4% was detected in 10 out of 13 (77%) iCCA cases positive for membrane hENT-1 expression, whereas only in 1 case (9.1%) in hENT-1 membrane negative patients (*p* = 0.0013). In addition, we observed that patients receiving adjuvant gemcitabine expressing mutant p53 cells > 4% in tumor tissue had a longer DFS than patients expressing mutant p53 cells ≤ 4% (median 18.5 vs. 6 months, *p* = 0.0229) (Figure 3).

### 2.2. hENT-1 Is Upregulated in Mutant p53 iCCA Cell Lines

Next, we examined the link between p53 status and hENT-1 expression in a panel of iCCA cell lines. To this purpose, basing on DepMap portal (https://depmap.org/portal/, accessed on 11 November 2024), wild-type p53 RBE, CCLP1, and SNU1079 cell lines, and mutant p53 HUCCT1, SSP25 (both carrying TP53 R175H mutation) and HUH28 (carrying TP53 E271K mutation) cell lines were chosen.

**Figure 4:**
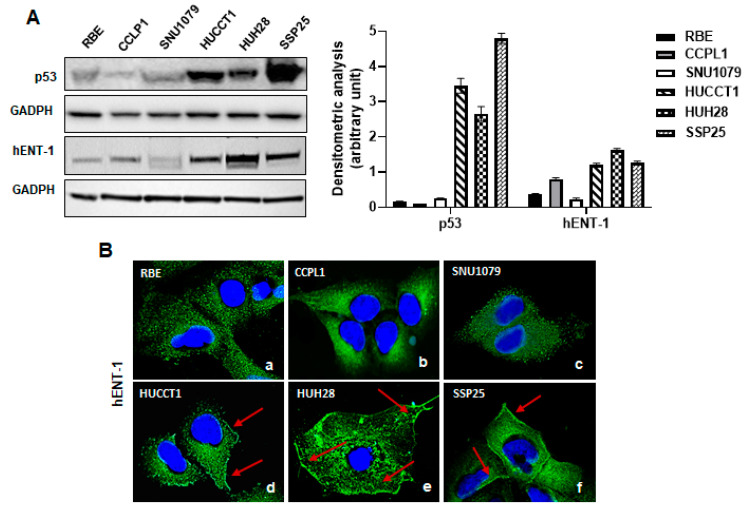
(**A**) Western blotting analysis of p53 and hENT-1 expression in wild-type p53 RBE, CCLP1, and SNU1079 and mutant p53 HUCCT1, HUH28, and SSP25 iCCA cell lines. GADPH was assessed as a quantitative control for equal loading. Histograms show densitometric analysis of p53 and hENT-1 protein expression normalized to corresponding GADPH level. (**B**) Confocal microscopy analysis of hENT1 localization in wild-type p53 RBE, CCLP1, and SNU1079 (a–c) and mutant p53 HUCCT1, HUH28, and SSP25 (d–f) iCCA cell lines. Red arrows show positive membrane expression in mutant p53 HUCCT1, HUH28, and SSP25 iCCA cell lines. Hoechst 33342 was used for nuclear staining. Magnification 60×.

As shown in Figure 4A, Western blotting analysis showed that mutant p53 HUCCT1, SSP25, and HUH28 cells expressed higher levels of p53 protein compared to RBE, CCLP1, and SNU1079 wild-type p53 cells. These findings were in line with previous reports showing that in cancer cells, mutant p53 protein is detected at higher levels than the wild-type form, as the last has a short half-life due to constitutive degradation by the ubiquitin ligase MDM2 [11]. Moreover, similarly to what was observed in iCCA tissue samples, we found that hENT-1 expression was higher in mutant p53 cell lines compared to wild-type p53 cells (Figure 4A). Further confocal analysis revealed a diffuse hENT-1 cytoplasmic dot-like staining in RBE, CCLP1, and SNU-1079 wild-type p53 cells, and a strong cytoplasmic/membrane positivity in HUCCT1, SSP25, and HUH28 mutant p53 cell lines (Figure 4B(a–f)), suggesting a different hENT-1 subcellular distribution between mutant and wild-type p53 iCCA cell lines.

### 2.3. p53 Status Regulates hENT-1 Expression in iCCA Cell Lines

**Figure 5:**
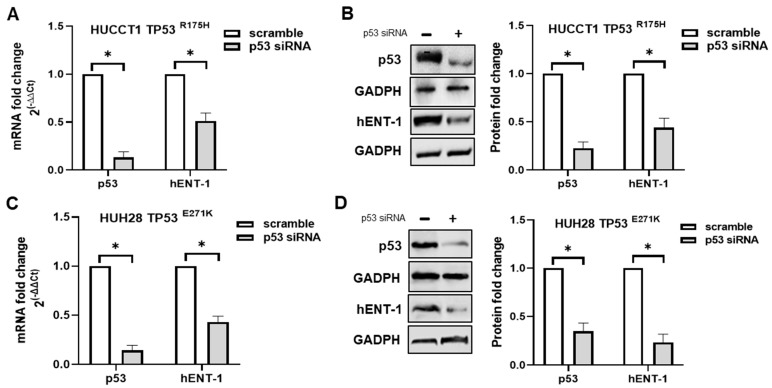
(**A**,**C**) p53 and hENT-1 mRNA fold change in HUCCT1 and HUH28 mutant p53 cell lines transfected with p53 siRNA and scramble for 48 h. * *p* < 0.05. (**B**,**D**) Western blotting analysis of p53 and hENT-1 protein expression in HUCCT1 and HUH28 mutant p53 cell lines transfected with p53 siRNA and scramble for 48 h. GADPH was assessed as a quantitative control for equal loading. Histograms show densitometric analysis of p53 and hENT-1 protein expression normalized to corresponding GADPH level. * *p* < 0.05.

To provide further evidence about the link between p53 status and hENT-1 expression, mutant p53 was transiently knocked down in HUCCT1 and HUH28 cells. As shown in Figure 5A–D**,** a significant decrease in p53 mRNA and protein expression was observed in both cell lines after 48 h from p53 siRNA transfection, confirming effective gene silencing. Concomitant analysis of hENT-1 mRNA and protein expression demonstrated that they were significantly decreased in p53-silenced cells compared to scramble (Figure 5A–D).

**Figure 6:**
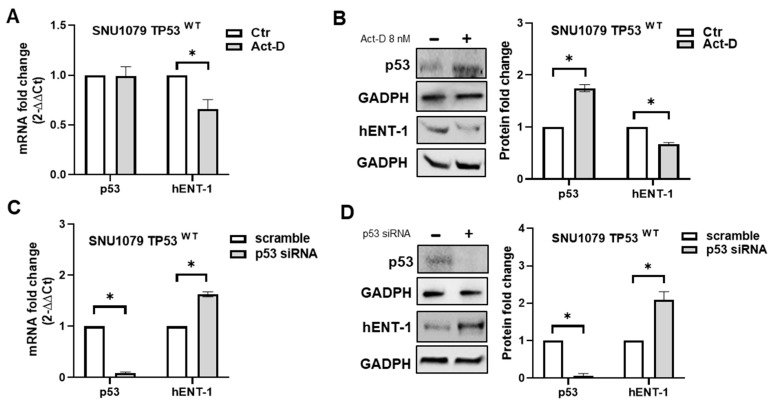
(**A**) p53 and hENT-1 mRNA fold change in wild-type p53 SNU1079 cells treated for 8 h with Act-D 8nM. * *p* < 0.05. (**B**) Western blotting analysis of p53 and hENT-1 protein expression in wild-type p53 SNU1079 cells treated for 8 h with Act-D 8nM. GADPH was assessed as a quantitative control for equal loading. Histograms show densitometric analysis of p53 and hENT-1 protein expression normalized to corresponding GADPH level. * *p* < 0.05. (**C**) p53 and hENT-1 mRNA fold change in wild-type p53 SNU1079 cells transfected with p53 siRNA and scramble for 48 h. * *p* < 0.05. (**D**) Western blotting analysis of p53 and hENT-1 protein expression in wild-type p53 SNU1079 cells transfected with p53 siRNA and scramble for 48 h. GADPH was assessed as a quantitative control for equal loading. Histograms show densitometric analysis of p53 and hENT-1 protein expression normalized to corresponding GADPH level. * *p* < 0.05.

Next, in order to assess whether hENT-1 expression could be repressed by endogenous wild-type p53, SNU1079 cells were treated for 8 h with Actinomycin-D (Act-D) 8nM, a drug able to stabilize and restore wild-type p53 function by induction of ribosomal stress and inhibition of the p53 negative regulator MDM2 [12]. No significant difference in p53 mRNA level was observed in SNU1079 cells treated with Act-D compared to controls (Figure 6A); conversely, this drug significantly increased wild-type p53 protein expression and accumulation in treated cells (Figure 6B). These findings confirm previous studies showing that low concentrations of Act-D are able to stabilize wild-type p53 protein without increasing gene transcription [12]. When we analyzed hENT-1 levels, both mRNA and protein expression were decreased in cells treated with Act-D compared to controls (Figure 6A,B). As a further control, SNU1079 cells were transiently transfected with p53 siRNA; as shown in Figure 6C,D, hENT-1 mRNA and protein expression were induced in p53-silenced cells compare to scramble. Overall, these findings suggest a role of p53 status in regulating hENT-1 expression in iCCA cancer cells.

## 3. Discussion

In recent years, the prognostic and predictive role of the nucleoside transporter hENT-1 has emerged in different cancer types and clinical settings [4,5,13,14,15,16,17]; nevertheless, the molecular mechanisms regulating its expression in cancer patients have been less investigated.

A downregulation of hENT-1 expression has been previously reported in response to inflammatory cytokines and following activation of the hypoxia-activated transcription factor HIF1α and JNK-cJun signaling cascade [18,19,20].

In the present study, we report a novel mechanism underlaying hENT-1 regulation that is linked to p53 status in cancer cells. In iCCA cell line models, a higher hENT-1 expression was detected in mutant p53 cells compared to wild-type p53 cells; moreover, in mutant cell lines, this nucleoside transporter was localized not only on the cytoplasm, but also on the cell membrane of cancer cells. Transient knockdown of mutant p53 in iCCA cells carrying different TP53 mutations (R175H and E271K) resulted in a decrease of hENT-1 mRNA and protein levels in both cell line models. On the other hand, in wild-type p53 cells, hENT-1 mRNA and protein levels increased after p53 silencing, while their decrease was observed after stabilization and restoration of wild-type p53 function by Act-D. Overall, these findings suggest an opposite function (activating versus repressive) of mutant and wild-type p53 protein on hENT-1 regulation. This observation agrees with previous studies reporting that several genes involved in nucleotide de novo synthesis and salvage pathways are targets of mutant p53 proteins, and that their expression (both at mRNA and protein level) positively correlates with the levels of mutant p53 [10]. Indeed, as mutant p53 cells show an uncontrolled proliferative stimulus, the upregulation of these genes is required to increase the intracellular nucleotide pool to sustain RNA/DNA biosynthesis and cellular bioenergetics [9]. In this scenario, the increased expression of hENT-1 detected in mutant p53 iCCA cells compared to wild-type p53 cells is therefore biologically conceivable.

The positive association between mutant p53 cells and hENT-1 expression was also observed in tumor tissue of iCCA patients; moreover, patients with mutant p53 cells > 4% showed a higher localization of the transporter on cancer cell membrane compared to patients with mutant p53 cells ≤ 4%. This finding could explain why, when these patients received adjuvant gemcitabine, those expressing mutant p53 cells > 4% showed a longer DFS than patients expressing mutant p53 cells ≤ 4%. Indeed, as we previously reported, an efficient uptake of gemcitabine by cancer cells requires proper hENT-1 localization on the cell membrane, and aberrant localization of this transporter in cancer cells may result in a decreased/lack of response in patients receiving this drug [4]. The better response to adjuvant gemcitabine in iCCA patients expressing a higher number of mutant p53 cells in the tumor tissue is in line with the findings of the CONKO-001 trial. In this phase III randomized study, evaluating the efficacy of adjuvant gemcitabine in resected pancreatic cancer compared to an observational group, TP53 mutations represented a negative prognostic factor for DFS in untreated patients (HR: 2.434, *p* = 0.005). However, in patients receiving gemcitabine, TP53 mutations were associated with a better DFS compared to patients with wild-type p53 (HR: 0.235, *p* < 0.001 in mutant p53 patients; HR: 0.794, *p* = 0.483 in wild-type p53 patients), with a significant test for interaction (*p* = 0.003) [21]. It can be hypothesized that the benefit from adjuvant gemcitabine in pancreatic cancer patients carrying TP53 mutations could be related, at least in part, to a concomitant increased expression and membrane localization of hENT-1 in tumor tissue, similarly to what we observed in iCCA patients.

## 4. Materials and Methods

### 4.1. Patients

Thirty-eight consecutive iCCA patients who underwent surgery with curative intent and received adjuvant gemcitabine at our Center from May 2014 to May 2024 were included in this study. Data on clinical variables, including sex, age, and tumor/node/metastasis classification, were gathered from patients’ records. Morphological classification of the tumors was based on the World Health Organization criteria [11]. Tumors were graded according to the international tumor node-metastasis (TNM) system [12].

Patients were considered suitable for adjuvant gemcitabine chemotherapy according to the inclusion criteria reported in our previous study [4]. The adjuvant chemotherapy schedule consisted of gemcitabine monotherapy 30 min intravenous infusion at 1000 mg/m^2^ on days 1, 8, and 15 of a 28-day cycle for 6 cycles, as previously reported [4]. All patients started the first infusion between days 30 and 60 after surgery and the overall duration of chemotherapy was 6 cycles for 6 months.

### 4.2. Tissue Microarray and Immunohistochemistry

Tissue microarray (TMA) was built from formalin-fixed, paraffin-embedded tissue samples from iCCA patients; for each case, three representative neoplastic cores were assembled in the TMA block. Immunohistochemistry (IHC) analysis was performed on serial 3 µm thick sections with anti-hENT-1 (GeneTex, Inc., San Antonio, TX, USA) and anti-mutant p53 (clone E47) (Abcam; Cambridge, UK) primary antibodies. IHC for hENT-1 was carried out as previously reported [4,5]. IHC for mutant p53 was performed using a Benchmark Ultra immunostainer (Ventana/Roche) according to the following protocol: (a) dewaxing; (b) antigen retrieval in Cell Conditioning 1 for 36 min at 95 °C; (c) incubation with primary antibody for 32 min at 37 °C; (d) development using the UltraView Alkaline Phosphatase Red detection kit and counterstaining in hematoxylin. Mutant p53 was considered overexpressed when mean percentage of positive nuclei in three TMA cores was >10%. IHC analysis was performed by two dedicated pathologists blinded to each other and to patients’ clinical data.

### 4.3. Cell Lines

The human iCCA cell lines SNU1079, HUCCT1, HUH28, CCPL1, RBE, and SSP25 were obtained from the Korean Cell Line Bank-KCLB (Seoul, Republic of Korea) and Dr. Chiara Braconi (University of Glasgow, Glasgow, Scotland). Cells were cultured in RPMI 1640 medium (Euroclone, Milan, Italy), supplemented with 10% (*v*/*v*) heat-inactivated fetal bovine serum (FBS), 2 mM L-glutamine, 100 U/mL penicillin, and 100 µg/mL streptomycin (Euroclone). Cells were grown at 37 °C in a humidified atmosphere of 95% air and 5% CO_2_ and routinely passaged using trypsin-EDTA 0.025% (Euroclone).

### 4.4. p53 siRNA Transfection

For TP53 gene silencing, the TriFECTa RNAi Kit from IDT (Integrated DNA Technologies, Coralville, IA, USA) was used according to the manufacturer’s instructions. Briefly, SNU1079 (2 × 10^5^), HUH28 and HUCCT1 (1.5 × 10^5^) cells were seeded in a six-well plate in antibiotic-free medium. Transfection was performed with Lipofectamine (Invitrogen, Waltham, MA, USA) in Opti-MEM medium (Invitrogen), using a pool of siRNAs (Invitrogen) targeting different regions of p53 mRNA at a concentration of 0,5 µg/well for 4 h. At the end of incubation, the medium was replaced with fresh antibiotic-free growth medium. In each experiment, control cells were transfected with equal amounts of non-specific siRNA control (scramble). RNA and proteins were extracted at 48 h after p53 siRNA transfection.

### 4.5. RNA Isolation and qRT-PCR

Total RNA was isolated from iCCA cell lines using the AllPrep DNA/RNA Mini Kit (Qiagen, Valencia, CA, USA) according to the manufacturer’s instructions. A total of 0.5 µg of RNA was reverse-transcribed using ImProm-II Reverse Transcriptase (Promega Corporation, Madison, WI, USA), following the manufacturer’s protocol. Primers for SYBR Green qRT-PCR analysis of p53 mRNA were purchased from Applied Biosystems (Carlsbad, CA, USA). For these primers, the following cycling conditions were applied: 50° for 2 min; 95 °C for 10 min followed by 40 cycles of 95 °C for 15 s and 60 °C for 1 min. hENT-1 and glyceraldehyde 3-phosphate dehydrogenase (GAPDH) mRNA expression was analyzed by quantitative reverse-transcriptase polymerase chain reaction using TaqMan Gene Expression Assays (Applied Biosystems, Carlsbad, CA, USA). Fold change in p53 and hENT-1 mRNA expression was calculated according to the 2^−∆∆Ct^ method.

### 4.6. Western Blotting Analysis

Western blotting was performed as previously reported [5] with the following primary antibodies: anti-p53 (Novocastra, Newcastle, UK) recognizing both wild-type and mutated p53 protein, anti-hENT-1 (Abcam), and anti-GADPH (Cell Signalling, Danvers, MA, USA). GADPH was used for equal protein loading. Digital images of X-ray films will be captured by Invitrogen iBright Imaging Systems (Invitrogen).

### 4.7. Confocal Microscopy

SNU-1079, HUCCT-1, HUH28, CCPL-1, RBE, and SSP25 cell lines were plated on glass coverslips and fixed with 4% paraformaldehyde in phosphate-buffered saline (PBS) for 20 min. Permeabilization and blocking were performed in PBS with 10% FBS and 0.1% saponin. Primary hENT-1 antibody was from Thermo Fisher Scientific and secondary Alexa Fluor conjugated antibody 488-goat anti-rabbit IgG from Invitrogen. DNA was stained with 1 µg/mL Hoechst 33342 for 5 min at room temperature. Coverslips were mounted using 0.5% p-phenylenediamine in 20 mM Tris 8.8 and 90% glycerol. Slides were analyzed with confocal Nikon Eclipse 80i microscope (Nikon Instruments Italia, Firenze, Italy).

### 4.8. Statistical Analysis

*t*-Student and ANOVA tests were used for comparison of continuous variables between two or more groups, as appropriate; categorical variables were compared with Fisher’s exact test. Cohen’s k was used for interobserver agreement for hENT-1 IHC analysis. Receiver operating characteristic (ROC) curve was built to assess mutant p53 cell cut-off among groups, and the area under the curve (AUC) was calculated. Kaplan–Meier survival curve with log-rank test was used to test differences in disease-free survival (DFS) between groups. DFS was defined as the time from the date of iCCA surgery to the date of relapse, death from any cause, or last follow-up. All analyses were performed using GraphPad Prism 9.3.1 software version (GraphPad Software, San Diego, CA, USA). A *p*-value < 0.05 was considered statistically significant.

## 5. Conclusions

In conclusion, here we provide new insights about the molecular mechanisms of hENT-1 regulation in iCCA, showing a link between increased mutant p53 and increased hENT-1 expression/membrane localization in cancer cells. Furthermore, our findings suggest a possible predictive role of p53 status in iCCA patients treated with gemcitabine; however, due to the small patients’ population included in the present study, the results should be considered as preliminary, and need to be confirmed in further investigations on a larger patients’ population.

## Figures and Tables

**Table 1:** Baseline characteristics of the 38 iCCA patients included in this study.

**Characteristics**	**Patients (*n* = 38)**
Age at surgery (years), median (range)	61 (33–79)
*Gender, n (%)* females males	20 (52.6) 18 (47.4)
*Histological grade, n (%)* G1 G2 G3 G4 NA	9 (23.7) 8 (21) 13 (34.2) 3 (7.9) 5 (13.1)
*Size and extent (T), n (%)* T1 T2 T3 T4 NA	11 (29) 19 (50) 4 (10.5) 4 (10.5) 0 (0)
*Regional lymph nodes (N), n (%)* N0 N1 NA	20 (52.6) 14 (36.8) 4 (10.6)
*Distant metastases (M), n (%)* M0 M1 NA	36 (94.7) 2 (5.3) 0 (0)
*Resection margins, n (%)* R0 R1 NA	37 (97.3) 1 (2.7) 0 (0)

**Table 2:** Correlation between hENT-1 membrane localization and mutant p53 cells in the tumor tissue from 38 iCCA patients included in this study.

	**Mutant p53 Cells ≤ 4%**	**Mutant p53 Cells > 4%**	*p*-Value *
Membrane hENT-1 positive	3	10	0.0013
Membrane hENT-1 negative	10	1	

* Fisher’s exact test.

## Data Availability

All data generated or analyzed during this study are included in this published article.
